# Organizational culture and barriers to change in University of Gondar Comprehensive Specialized Hospital Cardiac Unit

**DOI:** 10.1186/s12913-023-09278-x

**Published:** 2023-03-28

**Authors:** Missaye M Mengstie, Gashaw A Biks, Emily J Cherlin, Leslie A Curry

**Affiliations:** 1 Department of Psychology, College of Social Sciences and the Humanities, University of Gondar, P.O. Box 196, Gondar, Ethiopia; 2 Institute of Public Health, College of Medicine and Health Sciences, University of Gondar, Gondar, Ethiopia; 3 Global Health Leadership Initiative, Yale School of Public Health, New Haven, CT USA; 4 Yale School of Management, New Haven, CT USA

**Keywords:** Organizational culture, Cardiac care, Culture change, Barriers, Hospital

## Abstract

**Background:**

Cardiovascular disease is a major contributor to high mortality in Ethiopia. Hospital organizational culture affects patient outcomes including mortality rates for patients with cardiovascular disease. Therefore, the purpose of this study was to assess organizational culture and determine barriers to change in the Cardiac Unit of University of Gondar Comprehensive Specialized Hospital.

**Methods:**

We used a mixed methods approach with a sequential explanatory design. We collected data through a survey adapted from a validated instrument measuring organizational culture (n = 78) and in-depth interviews (n = 10) with key informants from different specialty areas. We analyzed the quantitative data using descriptive statistics and the qualitative data through a constant comparative method of thematic analysis. We integrated the data during the interpretation phase to generate a comprehensive understanding of the culture within the Cardiac Unit.

**Results:**

The quantitative results indicated poor psychological safety and learning and problem solving aspects of culture. On the other hand, there were high levels of organizational commitment and adequate time for improvement. The qualitative results also indicated resistance to change among employees working in the Cardiac Unit as well as other barriers to organizational culture change.

**Conclusion:**

Most aspects of the Cardiac Unit culture were poor or weak, signaling opportunities to improve culture through identifying culture changing needs, implying the need to be aware of the subcultures within the hospitals that influence performance. Thus, it is important to consider hospital culture in planning health policy, strategies, and guidelines.

**Recommendations:**

It is of paramount importance to strengthen organizational culture through fostering a safe space that enables workers to express divergent views and actively considering such views to improve the quality of care, supporting multidisciplinary teams to think creatively to address problems, and investing in data collection to monitor changes in practice and patient outcomes.

## Background

Organizational culture is important to increase performance of hospitals and other healthcare centers [1, 2]. Positive organizational culture that emphasizes collaboration and teamwork contributes to improve healthcare services, patient satisfaction, and implementation of new innovations [3]. Some cultures facilitate improvement, support to generate new ideas and motivate employees to attempt for organizational success [4] while others defend to maintain the status quo [5] and negatively affect organizational reforms [6].Thus, it is of paramount importance to encourage cultures that can help organizations to be strong and competitive [7].

Cardiovascular disease causes high mortality in Ethiopia [8, 9]. Despite this, the Health Policy of Ethiopia, and the Health Sector Transformation Plan [10] and the Health Sector Development Program IV of Ethiopia [11] mainly focus on expansion of health infrastructures and human resources, ignoring investments in improving healthcare culture that can promote attainment of goals. Even though there is increased global awareness on the impact of organizational culture in hindering and supporting the success of healthcare centers, there is a scarcity of studies in low-income countries [3].

In Ethiopia, the existing studies focused on impact of organizational culture on effectiveness of public higher institutions [12], management innovation [13], information system in public and private sectors [14], information security compliance [15], job satisfaction [16]. A study by Mesfin [16] shows positive relationship between organizational culture and job satisfaction among health workers in Jimma zone and Jimma town administration hospitals. However, the previous studies did not investigate existing organizational culture along with exploring its impacts on organizational effectiveness. In addition, researchers, policymakers, and practitioners in Ethiopia have given limited attention to study hospital organizational culture and barriers to desired changes. Therefore, this study mainly examined organizational culture to provide foundational insights into organizational culture to inform healthcare policies, and initiatives, or studies and to add a unique perspective to the scholarship on organizational culture in which low-income countries are largely underrepresented. To this end, this study aims to: (1) assess organizational culture of the cardiac unit in University of Gondar comprehensive specialized hospital, and (2) explore barriers to changing culture.

## Methods

### Study setting

Since cardiovascular disease is the leading communicable disease that cause high disease burden in Ethiopia [8, 9], it deserves particular research attention from policy makers, and pratitioners. This research was conducted in University of Gondar Comprehensive Specialized Hospital Cardiac Unit.

The hospital is one of the teaching hospitals in Ethiopia and provides cardiac service under chronic outpatient department (OPD). The cardiac services is provided in one building with other OPD patients including diabetes and hypertension patients who share the same waiting area. After diagnosis, cardiac patients continue their follow-up once in a week.

### Study design

This study employed a mixed methods sequential explanatory design, combining quantitative data (e.g., questionnaires) and qualitative data (e.g., in-depth interviews), as appropriate to assess organizational culture [17].

### Participants

Quantitative survey: All the eighty health workers (54 males and 26 females) who provided cardiac care services in the hospital for at least one year were invited to complete a survey instrument to assess organizational culture in the cardiac unit. However, two participants did not complete the survey. Thus, the responses from seventy-eight participants including Nurses, Pediatric specialists, Resident Doctors (RD), General Practitioners (GP) and Intern doctors were used for analysis.

Qualitative interviews: Ten healthcare workers (8 male and 2 female) who provided cardiac care service for at least for one year in the center were purposively selected from different specializations.

### Data collection

Survey instrument: Quantitative data were collected using a scale adapted to measure organizational culture in Ethiopia’s primary health care system by Liu and colleagues [18]. The instrument consists of 26 items with five response options ranging from strongly disagree (1) to strongly agree (5) for measuring organizational culture in primary healthcare in Ethiopia. The tool has Cronbach’s alpha value of 0.84 that shows acceptable internal consistency [18]. We also checked and found a Cronbach’s alpha value of 0.78 indicating above the acceptable internal consistency.

The instrument measures five domains of hospital organizational culture including: learning environment (12 items), psychological safety (4 items), organizational commitment (3 items), senior management support (3 items), and time for improvement (4 items).

There are various organizational culture dimensions that influence employees’ performance such as involvement, collaboration, transmission of information, learning, care about clients, adaptability, strategic direction, reward and incentive system, control system, communication, agreement, and coordination and integration [19]. However, the present study focuses on assessing hospital organizational culture that includes learning and problem solving, psychological safety, time for improvement, resistance to change and commitment to organization. We chose this survey for two reasons: (1) it focuses on organizational culture in healthcare setting, and (2)  it measures broader domains of organizational culture as compared to other tools that focus on safety culture in low and middle income countries [18].

The learning and problem solving domain of hospital organizational culture encompasses a working environment that supports coordination between different departments, senior management involvement and support to improve service, and availability of the required personnel and equipment to provide healthcare [18]. Psychological safety refers to the extent to which employees are encouraged and allowed to discuss problems and different issues, freedom to freely express concerns and opposing views and value to new ideas [18]. It can be shaped by a wide range of factors such as authoritarian leadership behaviors, generational and other inter-group differences, and hierarchical nature of medicine. Resistance to change measures whether employees resist new approaches and policies [18]. Time for improvement refers whether the working environment makes employees too busy for improving service, the degree of stress, and the amount of time to review work processes. Finally commitment to organization measures feeling of emotional attachment and sense of belongings of employees to the organization [18].

Cognitive interviews were conducted with 5 healthcare workers in University of Gondar Comprehensive Specialized hospital to assess understanding and comprehensiveness of items. After the cognitive interviews, the wordings of four items were modified and simplified to be easily understood by participants.

In-depth interviews: The qualitative data were collected using eleven semi-structured interview questions. Sample items include: What is it like to work in cardiac service unit? How do you describe commitment of employees? How do people communicate? How would you describe the level of vertical and horizontal communication? How comfortable are people in speaking up to offer a different view? How does the senior management show support for the staff? We conducted interviews until data saturation. The interviews took approximately 2 hours.

### Data analysis techniques

Quantitative data: Descriptive statistics (percentage and mean) were used to compute demographic characteristics and organizational culture. Frequencies and percentages were used to summarize demographic information of the participants. The mean and standard deviation for the five subscales were computed to describe each domain of organization culture at University of Gondar Comprehensive Specialized Hospital Cardiac Unit. We calculated mean scores on each dimension. The mean of the scale (3) was used as a cut off value. A mean score greater than 3 on each subscale indicates strong or positive hospital organizational culture where as a mean score less than or equal to 3 indicates low or weak organizational culture.

Qualitative data: The qualitative data analysis passed through series of procedures. Initially, audio-recorded interviews were listened repeatedly. Then, the researcher transcribed the recorded audio data. The Amharic transcripts were again translated into English. After translation, codes were created based on research questions. From categorized codes, themes were generated and analyzed through thematic analysis technique. The researcher (Missaye Mengstie) along with two co-authors (Leslie Curry, Emily Cherlin) used the constant comparative method [20–22] iteratively comparing coded transcript segments to identify novel concepts, ensure consistent identification of emerging themes, and expand or refine codes.

## Results

This section presents qualitative and quantitative findings. The study sample included participants for survey and interviewees representing a diverse specializations and roles. Table 1 describes the roles of participants.

**Table 1:** Survey and in-depth interview participants

Role	Survey participants		In-depth Interview participants	
	n	%	n	%
Nurse	38	48.7	2	20
Specialist Doctor	7	9.0	2	20
Resident Doctor	10	12.8	1	10
General practitioner	13	16.7	1	10
Intern Doctor	10	12.8	1	10
Senior management	-	-	1	10
Radiologist	-	-	1	10
Porter	-	-	1	10
Total	78	100	10	100

### Quantitative results

One objective of this study was to assess organizational culture of the cardiac care unit. Table 2 shows means and standard deviations on each organizational culture dimension. Low mean scores indicated poor or weak organizational culture whereas high mean scores show strong organizational culture. The results indicated poor or weak overall organizational culture (Mean = 2.91, SD = 0.41). More specifically, low mean scores were found on learning and problem solving (Mean = 2.64, SD = 0.65), and psychological safety (Mean = 2.64, SD = 0.65) dimensions of organizational culture. These results indicated poor organizational culture in learning and problem solving and psychological safety aspects of organizational culture. However, participants reported high mean scores on time for improvement (Mean = 3.31, SD = 0.83), commitment to organization (Mean = 3.34, SD = 0.93) and resistance to change (Mean = 3.33, SD = 0.73) domains. A high mean score in these domains reveals strong sense of belongingness, availability of adequate time for improvement but a high resistance to change.

**Table 2:** Organizational culture in the cardiac care unit

Organizational culture dimensions	N	Mean	Std. Deviation
Learning and Problem Solving	78	2.64	0.65
Psychological Safety	78	2.62	0.58
Resistance to Change	78	3.33	0.73
Time for Improvement	78	3.31	0.83
Commitment to the Organization	78	3.40	0.93
Overall organizational culture	78	2.91	0.41

### Qualitative results

#### Learning and problem solving

The major themes that emerged under learning and problem solving aspect of the hospital culture focus on senior management support, vertical and horizontal communication, partiality, and working environment ( working space, medicine, facilities and equipment).

#### Senior management support

The engagement of the senior management to prioritize cardiac healthcare and to improve the service was not encouraging. The management’s attempt to solve personnel, material and equipment constraints was minimal. The participants of this study described that the senior management gave little attention to cardiac care services. A medical student in internship expressed his feelings as follows.

*Sometime it is difficult. There are many things to be improved. Top officials also know this. Though the country in general is not in good condition, there are things that need attention from the management (ID_08).*

Similarly, another interviewee (Radiologist) said that the engagement and support from higher officials is minimal and the effort to improve cardiac care was not at desired level. In support of this, a child pediatrician witnessed the negligible interest and engagement of the senior management to furnish adequate medical equipment, and facilities for cardiac patients. However, two of the interviewees (both nurses) appreciated the plan of the senior management to open cardiac clinic, to resolve room scarcity and to fulfill human resources. Similarly, an interviewee from senior management reported that the senior management is trying to put utmost effort to improve the cardiac service.

#### Horizontal communication

Participants were asked about their relationship with co-workers in their unit and across departments. Most participants reported presence of positive, smooth and friendly relationship between the healthcare staff in charge of providing cardiac service and the healthcare staff in others departments. A General Practioner (GP) doctor described staff collaboration as follows:

*Regarding staff coordination, sometimes there is no even division of work. The nurse may sometimes work as a porter. I am happy in this regard. We think for the patients because if there is delay due to shortage of porter, the service for patients will be delayed (ID_06)*.

The collaboration at internal medicine is generally good, and they support each other. However, there was relationship gap between Physicians and Pharmacists. One Resident Doctor (RD) expressed this problem as follows:

*In internal medicine, we work collaboratively. We also treat diabetic and hypertension patients. We work together with nurses. But there is relationship gap with pharmacists. They [pharmacists] don’t give us up-to-dated information about what is the available medicine, what new medicines arrived or which medicine is not available in the pharmacy (ID_07).*

#### Vertical communication

This study explored different relationships between subordinates and immediate supervisors or seniors. The relationship of senor medical doctors with Internist students and junior physicians was not based on mutual respect or professionalism. The senior doctors and immediate supervisors do not respect students in internship and junior doctors. This has been considered as a trend and tradition. One Intern expressed their relationship with supervisors as follows:

*To tell you the truth, there are seniors who harass us. Internship is a time we suffer. It is a trend and it continues. The seniors have passed through this trend and want to apply now. They expect from Intern students to pass through the hardship and there are abuses and insults from the seniors. Though it is the worst time we are facing , it has positive side in making us more resilient (ID_08).*

#### Partiality

The term called *“social*” is commonly used in daily activities in University of Gondar Comprehensive Specialized Hospital. *Social* means providing priority of service based on blood relationship, social connection, and friendship. Even if there are many patients awaiting the doctor, patients who have the so called social are treated first regardless of arrival time and number of patients. It is widely practiced in the cardiac care provisions. All of the interviewees admitted the practice of social in the hospital in general and in the cardiac unit as well. An interviewee (senior management) admitted the prevalence of healthcare service provision based on one’s social as follows:

*If a person [healthcare staff] claims treatment for everyone whom he/she knows, the hospital becomes a private entity rather than an institution established for the public good. I don’t mean I give favor for my social. This is the big problem not only in the cardiac department but also in the hospital in general. There should not be special treatment for being a social of a health staff. All patients should have been strictly treated based on seriousness of the problem (emergency first) then priority should be given to children, elders and mothers. But it is not possible to deny the presence of favoring and giving unfair treatment to one’s social. It is challenging to make correction and avoid treatment based on social. It has to be resolved (ID_04).*

A Porter expressed his observations on the issue of social in the cardiac department as follows:

*Social has been practiced and it is a tradition. For instance, a person who works in this hospital may claim that his/her “social” should get treatment first. Let me be the first to see the doctor. For instance,  a staff with a social may claim to be the first before a women who came early in the morning from rural area to get treatment, The staff with a social (either friend, family) is often from urban and does not pay much for transport. It is hard to imagine fair treatment not only for cardiac patients but also for all patients in the hospital. Getting treatment through one’s social is very common here (ID_03).*

However, two specialists considered social as a special benefit of a medical staff. One of them stated this as follows:

*I don’t deny the presence of social. The social is not only in the cardiac service. The public thinks that if one has no relative or a person whom he/she knows in the hospital, he/she cannot get treatment timely and properly. If you have a social, your chart will be at the first order among many charts. But what I do during my spare time is that I will go to office and examine a person whom I know, family, and a friend. If, for example, my friend’s family is sick and come to me to seek treatment, it is me who will examine. This is the benefits of a staff. I mean getting priority of services. But if there is a seriously ill cardiac patient, I will give the first chance to him/her. But under normal condition, my social will get the first chance to be examined and treated. We don’t gain financial benefits. As a university staff we don’t claim money but such kind of priority should be given to the staff (ID_05).*

However, all of the interviewees agreed that they first serve seriously sick patients before anyone else regardless of social relationship.

#### Working environment (working space, medicine, equipment and materials)

There is lack of space, medicine, equipment and materials to treat cardiac patients. For instance, follow –up service for adult cardiac patients is given with other chronic patients in one narrow floor and different patients wait for doctors in one area. A porter who works there described it as follows.

*Cardiac patients should have their own patient waiting area. So, there should be a mechanism for cardiac patients especially for elders to establish free and open space because, they may push each other and fall down on the floor(ID_03)*

There is no adequate room space for cardiac care. All the participants reported absence of convenient working space for cardiac patients. For example, an interviewee (nurse) stated that the cardiac patient share room with other chronic patients (e.g. diabetes). Similarly, another interviewee (Radiologist) described the need to establish a separate cardiology clinic for cardiac patients as follows.

*The radiology service is given to all patients [in the hospital]in one room. It is good to have separate radiology for cardiac patients. Cardiovascular patients need to be treated in better set up. They often wait their turn as other patients. Cardiovascular patients should get all the required services (e.g., ECG. Echo, X-ray and other services (ID_09)*.

One Resident Doctor (RD) described the shortage of medical supplies, equipment and materials as follows.

*We have no improved medicines, equipment, and materials to provide treatment for cardiac patients. We can`t get up-to-date medicine. We work [give service] by what we have at hand. For instance, all cardiac patients need INR [International Normalized Ratio] diagnosis once a month, but it is not being delivered here. The patients visit and pay high amount of money to private hospitals and clinics to get treatment (ID_07).*

#### Psychological safety

Most interviewees said that every healthcare staff who provides cardiac care service may express opposing views. Though there is no restriction to forward requests, reflect concerns, suggestions and opinions, the response from the management was often not welcoming. The management does not appreciate and welcome the voices of the staff though there is no restriction to express views. One Specialist stated it as follows:

*You can express your opinion, idea and feelings, but they [the management] suppress it. Expressing feeling alone does nothing unless it brings change. We often ask. There are many questions to the management. However, the requests, suggestion, concerns and opinion are being hold without solution (ID_05)*.

Similarly one General Practitioner stated that the management does not give attention to the voices of the staff as follows:

*There are people who can express their feelings. The response from the management is negative. They [the management] reply it is up to you. In order to reflect opposite views, you need to report to the senior and the management; but they ignore it. They, sometimes, make you not to speak your views (ID_06).*

The seniors do not encourage juniors’ ideas and do not allow issues to be discussed together. One Resident Doctor (RD) expressed this as follows:

*Regarding vertical relationship with supervisors, we must accept the order of those who are our supervisors. I try to forward my ideas but the decision is always made by a senior. The same is true for those who are under my supervision. They should accept what I say. You should accept order like a military. Not to take responsibility, we document everything what the senior orders. Otherwise, if the senior orders, it is accepted forgranted. (ID_07).*

In addition, one GP stated that the relationship of seniors with juniors and internship students in medicine is unique, disappointing and frustrating. He expressed his feeling as follows:

*You think that your immediate superior always tends to punish you. The relationship is not like an older and younger brother. The relationship is bad. Since long time, it has not been corrected. You should be seen inferior and act lower in front of a senior. If you ask me the worst time, it is a time if internship. (ID_06).*

#### Time for improvement

As mentioned before, the quantitative results revealed high mean score on time for improvemt, indicating enough time to review and improve cardiac care services. The qualitative results, however, yield mixed findings on this domain. A Specialist said that there is no special burden for those who only engage in cardiac unit. He stated this as follows:

*There is no special burden for those who only work for cardiac patients. The Residents are expected to examine 20 patients per day. Cardiac follow-up does not overload much. Not more than 4 or 5 persons come as new cases per day. I don’t think there is time burden for those who only engage in cardiac care (ID_10).*

Similarly, another interviewee (nurse) stated that there is no shortage of time and the number of staff is enough to cover the service. However, other interviewees (General Practitioner, Resident Doctor, Radiologist, Internship student) stated shortage of time to improve service quality which causes to make medical error. For instance, one GP expressed the time burden as follows:

*There is no doubt about shortage of time. During the day we are on work, but we spend the night in thinking and evaluating about how we treated our patients. Patients rush and push each other to get treatment because there are many patients. Due to this, we don`t give the adequate advise to patients. We rush to cover large number of patients and we also become exhausted. We do not give them adequate information and advice due to time pressure. Because many patients wait for our call. This results in rushing to cover and sometimes cause medication error (ID_06).*

#### Commitment to organization

The qualitative findings revealed that the majority of the staff who are involved in cardiac care had strong sense of belongingness, and they expressed their commitment to serve cardiac patients. A nurse expressed the commitment of the staff as follows:

*I am telling this without exaggeration. We are committed to give our utmost effort for the better treatment of cardiac patients. Even though there is scarcity of materials, we go to another place (unit) to undertake the examination because, most patients come from remote places. So, to treat the patients, we try our best. Even when there is no medicine in our store, there are workers who buy medicine from their own for patients. If you forget a material outside, there is a person who collects the material. We consider it [the hospital] as our home (ID_01).*

#### Resistance to change

Most of the interviewees believed that many workers in cardiac care unit resist changes when new approaches, policies and guidelines are endorsed. An interviewee from top management described presence of strong resistance from the staff when new guidelines come into work. He added that the staff does not accept new ideas, rather want to keep the status quo. He expressed it as follows:

*There is a strong resistance when new guidelines are in place. We, as a management, have tried to improve the cardiac services, but when new ideas come, the staff resists and prefers to keep the status quo. They don’t give ears to new things. There is a challenge and a problem to accept new working policies and guidelines. Many staff is not open-minded, at least, to test new guidelines. There are tendencies of “it has been the way we work and to continue as it has been operationalized. (ID_04).*

In support of this, a Specialist stated that the senior staff is especially resistant to changes. He stated it as follows:

*In general, we are not comfortable when new things are endorsed. People fear changes. The senior staff is change resistant. They want the status quo to continue. The youngest staff, however, wants and appreciates to see new working condition. Obviously, there is resistance (ID_05)*.

#### Barriers to changing culture

This study identified different potential barriers to change the culture in the cardiac unit. The barriers can be group as organizational and personal barriers.

#### Organizational barriers

The participants mentioned various organizational factors that can be an obstacle for changing the culture of the cardiac unit. These include senior staff turnover, shortage of working space, lack of equipment, lengthy and bureaucratic purchasing and procurement process, and lack of attention from the senior managements. Participants described high personnel turnover, especially senior cardiologists as one of the main challenges to change the culture of the unit. All the interviewees worried about the increasing turnover rate of senior physicians. One pediatric specialist expressed his concern as follows.

*We had one adult cardiologist but he left [the hospital] in 2021. We had also two child cardiologists but one of them has already left. So, we had three radiologists (two child cardiologists and one adult cardiologist) before one year. Now we have just one cardiologist and we should pray to maintain him. When, we have no cardiologist at specialty level, a medical doctor who is interested in the area gives cardiac service by referring books (ID_ 05).*

Overall high senior staff turnover, lack of room space, lack of adequate medicine and equipment were identified to be hindering organizational factors to change the culture of the cardiac unit.

#### Personal barriers

The personal factors which can hinder culture changing were mentioned by the participants. These include senior staffs’ attitude, conflict of interest and engagement of medical doctors in their own private clinic. For example, one interviewee (nurse) described some of the major challenges that constrain culture change in the unit as follows:

*The first challenge is shortage of professionals. We need many healthcare workers. There is also scarcity of supportive staff. In addition, the work space should be more accommodative and convenient. Our medical doctors have their own clinics outside the hospital in the city with better treatment but with high payment. As a result, sometimes people from the city (Gondar) prefer and visit private clinics and hospitals despite a costly charge  (ID_01).*

Related to attitude of senior staff, one interviewee (pediatrics specialist) expressed that those who worked for very long time [seniors] want the status quo to be maintained, in contrast to the young and energetic staff who want to improve services.

In addition, there is conflict of interest between radiologists and cardiologists that can be considered as a barrier for culture change. One interviewee (radiologist) explained it as follows:

*The main challenge to improve radiology service to cardiac patients is conflict of interest. Our radiology unit claims to do Echo but the cardiologists also claim to do it. In the process, the patients stay long and receive delayed service. They [cardiologists] say that doing Echo is their task (ID_09)*.

In general, the current study revealed both organizational and individual factors that can be a barrier to organizational culture change.

## Discussion

The results of this study revealed challenges in the working environment to provide clinical cardiac care services in the University of Gondar Comprehensive Specialized Hospital, including high personnel turnover and staff shortages, lack of working space, and lack of equipment and medication. In addition, lack of room space for patients to wait, scarcity of medicine, equipment and materials are persistent challenges. Many previous studies in Ethiopia [23–25] also indicated high turnover intention of healthcare workers, and it is warning indicator of staff shortages. As the present study revealed, the turnover of senior cardiologists that affects the quality of cardiac services delivery raised a concern in the hospital. Turnover of senior doctors is exacerbated by national instability, poverty and conflict [24] as well as learning opportunities in other countries that are pulling factors for the exodus of health professionals from developing and Sub-Sahara Africa to high income countries [26]. 

The attempt of the senior management to solve personnel, material and equipment constraints was minimal. Their engagement and support to improve cardiac care was not at expected level, and their involvement to provide adequate material supplies and coordinating human resources to serve cardiac patients were not adequate. There are opportunities for improvement using models to bring culture change in University of Gondar Comprehensive Specialized Hospital management such as “Leadership Saves Lives” which is an intervention model recently developed to change hospital organizational culture for better patient outcomes [27].

The present study showed mixed findings regarding availability of time for improvement. The quantitative results indicated availability of adequate time to review and revise work process. However, the qualitative findings showed that General Practitioners, Residents, Radiologists and intern medical students faced shortage of time to improve service quality which even caused to make medical errors. Similarly, previous studies showed that there was high workload which caused stress among intern medical students in teaching hospitals in Ethiopia [28]. Besides high workload was a cause for high burnout among physicians working in southern Ethiopia [29]. In addition to this, a systematic review and meta-analysis among health care professionals in Ethiopia indicated that more than half of the healthcare workers experienced occupational stress [30]. At the University of Gondar Comprehensive Specialized Hospital some staff are in charge of providing healthcare services, conduct research and provide community services. These duties would cause work burden and stress on health workers in the cardiac department as well.

The horizontal communication among healthcare staff who provide cardiac service across units and departments was often positive, smooth and friendly. There was generally good coordination across units and departments involved in cardiac care. However, coordination was not good between medical doctors and pharmacists on the type and availability of medicine to treat cardiac patients. There was also interest conflict between medical doctors and radiologists over doing radiology to cardiac patients.

The vertical communication across hierarchies based on seniority, status and position was limited and was not usually healthy. The relationship between senior medical doctors and intern medical students and junior medical doctors was not based on mutual respect. Similarly, a study on relationship between physicians and nurses in Ethiopian revealed poor communication [31]. The national culture of Ethiopia would influence organizational cultures and subcultures. Ethiopian is characterized by high power distance culture [32] where people often accept hierarchical order without questioning or asking justification. Due to direct influence of national high power distance culture of Ethiopia, employees often do not feel comfortable asking questions for clarifications and challenging ideas of higher positioned or status persons which makes relationships challenging to young and vibrant employees [33]. This culture would influence juniors and intern doctors to accept any orders of senior doctors.

Coming to psychological safety, the present study uncovered limited freedom to express voice in cardiac unit in the University of Gondar Comprehensive Specialized Hospital. The management does not appreciate the voices of the staff. The response of the management to staff views, requests, concerns, suggestions and opinions was not welcoming.

Impartiality in terms of serving all patients equally was not often practiced in the cardiac unit due to the so called *social*. Providing a priority and quality service based on social connection (e.g., friendship, and blood relations) was dominantly practiced in providing care services. Despite the Hippocratic Oath in Medicine and in spite of first come first serve logic and no matter how much is the number of patients awaiting the call of the doctor, patients who have previous connection with healthcare workers or who have relatives working in the hospital are treated first. Though treatment based on one’s social is against the medical oath and immoral, it is widely practiced in the cardiac care provisions. It is considered as a tradition and a special benefit of a medical staff which is a major cause for dissatisfaction and complain of many patients. Providing healthcare service based on social is also against health equity principle of “giving people what they need when they need it, and in the amount, they need” [34].

Providing service based on first come first served rule endorses equal opportunity of receiving healthcare services to every patient [35]. However, treating people based on social relationship is against the principle of justice [36]. On the other hand, it should be noted that the participants assured their firm stand to prioritize and care the sickest cardiac patient regardless of social connection. But under normal condition, they disclosed that they serve and let to be served a patient with some social connection. Doctors considered this as a special benefit or favor of a health worker, forgetting the promise and the swear they promised during their graduation. Most participants of this study expressed strong sense of belongingness, high commitment and strong organizational sense of ownership. However, there were some staff who loss interest and commitment, and who were unhappy because of different reasons and their coarse relationship with their supervisors.

The current study indicated some important suggestions to improve culture in various aspects within the cardiac unit. Changing organizational culture can be done through reshaping culture of different sub - groups across units and departments [37]. The major areas of cardiac care culture that need change are related to learning and problem solving (personnel turnover and staff shortages, lack of working space, lack of equipment, materials, and medication), time for improvement, psychological safety, partiality in providing cardiac service, senior management support, communication (especially vertical) and resistance to change.

Overall, the current study revealed organizational and personal challenges that potentially hinder organizational culture change. However, organizational culture change requires members of the organization to feel safe and empowered to initiate creative problem solving, challenge the status quo, and take advantage of a wide range of diverse expertise among all staff. Our findings showed a number of barriers to create this type of environment such as hierarchy, conflict of interest, resistance to change and lack of support from the senior management.

## Limitation of the study

This study has some limitations related to sampling and scope that should be considered in making conclusions about organizational culture which is a complex construct to understand and measure. First, we used purposeful sampling for the in-depth interviews [22], which does not allow for generalizability to a wider range of cardiac hospitals. Second, only two out of ten interviewees were female nurses that limit gender representation. Third, data were collected only through survey and in-depth interviews that limit collecting information from diversified tools.

## Conclusion

The present study uncovered the different aspects of hospital organizational culture in the University of Gondar Comprehensive Specialized Hospital cardiac care unit. Related to learning environment, health workers were not encouraged to do things in a better way, and they are not encouraged to come up with new ways of solving problems. On the other hand, the management tends to maintain the status quo. In addition, there was absence of good coordination among different units and departments which provide cardiac care service. Besides to this, there was no favorable working environment in terms of human and physical resources. Furthermore, there was alarming turnover of senior staff that lead to shortage of staff, scarcity of medical facilities such as medicine, equipment and materials.

The visibility, engagement and support of the senior management in the cardiac care service were limited. There was communication gap between the cardiac staff and senior management. The senior management was reluctant to hear the voices of the cardiac staff. There was also serious communication problem in the hierarchical relations between the senior staff and junior staff and intern medical staff which was not friendly, and professional. Even sometimes, disagreements existed in horizontal communication between physicians with pharmacists and radiologists.

The biggest issue related to fairness or equality of opportunity was treating cardiac patients based on social connection. It has been widely practiced in the cardiac unit that has been considered as unwritten culture, norm, tradition or trend. Despite partiality in providing services, the organizational commitment of cardiac staff was high. The majority of the cardiac staff developed sense of belongingness and had feeling of emotional attachments to the unit.

### Recommendations

#### Policy

This study provides information to health policy makers on organizational culture at the cardiac department in the University of Gondar Comprehensive Specialized Hospital. The findings clearly revealed salient elements that hinder effectiveness of employees which would eventually influence patient outcomes. Thus, it is important to consider and incorporate hospital culture and subcultures in planning health policy, strategies, and guidelines in low income countries like Ethiopia where organizational culture has not been given due attention in improving healthcare services and patient outcomes.

#### Practice

Hospital administrators and managers should be aware of the subcultures within the hospitals. The hospital management should plan changing the culture of the cardiac care unit for improved patient outcomes and organizational success. In addition, the hospital management should invest in efforts to strengthen the culture of the cardiac care unit by: (1) fostering a safe space to express divergent views (2) supporting multidisciplinary teams to think creatively to address problems, and (3) investing in data collection to monitor changes in practice and patient outcomes.

#### Future research

There is a need for comprehensive study to assess organizational culture in low-income countries where the issue of organizational culture is largely ignored.

## Data Availability

The data sets are available from the corresponding author up on request.

## Abbreviations

OPD: Outpatient Department
GP: General Practitioners
RD: Resident Doctor
